# The cyclooxygenase-2 selective inhibitor NS-398 does not influence trabecular or cortical bone gain resulting from repeated mechanical loading in female mice

**DOI:** 10.1007/s00198-012-1922-0

**Published:** 2012-02-14

**Authors:** T. Sugiyama, L. B. Meakin, G. L. Galea, L. E. Lanyon, J. S. Price

**Affiliations:** 1 Department of Veterinary Basic Sciences, The Royal Veterinary College, University of London, London, UK; 2 School of Veterinary Sciences, University of Bristol, Langford, Bristol, BS40 5DU UK

**Keywords:** Bone architecture, Cyclooxygenase-2 selective inhibitor, Exercise, Female, Mechanical loading

## Abstract

**Summary:**

A single injection of the cyclooxygenase-2 (COX-2) selective inhibitor NS-398 reduces bone’s osteogenic response to a single period of mechanical loading in female rats, while women taking COX-2 selective inhibitors do not have lower bone mass. We show that daily NS-398 injection does not influence bone gain from repeated loading in female mice.

**Introduction:**

Prostaglandins are mediators of bone cells’ early response to mechanical stimulation. COX-2 expression is up-regulated by exposure of these cells to mechanical strain or fluid flow, and the osteogenic response to a single loading period is reduced by COX-2 inhibition. This study determined, in female mice in vivo, the effect of longer term COX-2 inhibition on adaptive (re)modelling of cortical and trabecular bone in response to repeated loading.

**Methods:**

Nineteen-week-old female C57BL/6 mice were injected with vehicle or NS-398 (5 mg/kg/day) 5 days a week for 2 weeks. On three alternate days each week, the right tibiae/fibulae were axially loaded [40 cycles (7 min)/day] three hours after injection. Left limbs acted as internal controls. Changes in three-dimensional bone architecture were analysed by high-resolution micro-computed tomography.

**Results:**

In control limbs NS-398 was associated with reduced trabecular number but had no influence on cortical bone. In loaded limbs trabecular thickness and cortical periosteally enclosed volume increased. NS-398 showed no effect on this response.

**Conclusion:**

Pharmacological inhibition of COX-2 by NS-398 does not affect trabecular or cortical bone’s response to repeated mechanical loading in female mice and thus would not be expected to impair the functional adaptation of bone to physical activity in women.

## Introduction

Mechanical loading is the principal functional determinant of bone mass and architecture [1–3], and numerous studies have shown that prostaglandin signalling plays a key role in mechanotransduction, with cyclooxygenase-2 (COX-2) expression being rapidly up-regulated in both osteoblasts and osteocytes following exposure to fluid flow or mechanical strain in vitro [4–6]. Blocking prostaglandin production with indomethacin in experimental animals in vivo has repeatedly been shown to impair the osteogenic response to a single period of mechanical loading in cortical and trabecular bone [7–9]. Two studies in female rats have also shown that a single injection of the COX-2 selective inhibitor NS-398, 3 h prior to a single period of mechanical loading, reduces the osteogenic response in the cortex [9, 10]. However, the effect of more sustained COX-2 selective inhibition on the adaptive response to mechanical loading in cortical bone remains less clear and is unknown in trabecular bone. In the cortex, the osteogenic response to two episodes of mechanical loading in genetically modified female mice lacking COX-2 was not impaired [11]. This could be due to compensation for the complete absence of COX-2 over the animals’ life time, a response which is less relevant to the clinical situation using COX-2 selective inhibitors if similar compensation occurs over the comparatively shorter term.

This issue is important to resolve, especially in women who have a higher risk of fragility fractures associated with osteoporosis than men, because non-steroidal anti-inflammatory drugs (NSAIDs), including COX-2 selective inhibitors, are widely prescribed and a decrease in the skeletal response to physical activity would result in bone loss. Interestingly, a recent randomized controlled trial [12] did not find a suppressive effect of ibuprofen, a nonselective COX inhibitor, on hip areal bone mineral density (BMD) in premenopausal women who performed weight-bearing exercise for 9 months. Consistent with this finding, among the users of COX-2 selective inhibitors, hip areal BMD was normal in postmenopausal women using oestrogen replacement therapy and higher in those not using oestrogen replacement therapy [13]. These clinical data appear to imply that functional adaptation of bone to daily loads is not inhibited by COX-2 selective inhibitors in women.

In the present study, we assessed whether NS-398 affects bone’s response to repeated periods of mechanical loading in female mice using the well-characterized non-invasive tibia/fibula axial loading model [14–16]. This model allows examination of the effect of local mechanical stimulation, distinct from that of exercise, in both trabecular and cortical bone compartments. To our knowledge, this is the first study investigating the effects of a COX-2 selective inhibitor on trabecular and cortical bone’s adaptive response to repeated periods of mechanical loading.

## Materials and methods

### Experimental design

The experiment was conducted in July–August 2009 at the Royal Veterinary College (London, UK), with the approval of the relevant ethical committees. Nineteen-week-old female C57BL/6 mice (Charles River Laboratories, Inc., Margate, UK) were divided into two body weight-matched groups (*n* = 8 in each group) and treated with subcutaneous injections of vehicle [dimethyl sulphoxide (2.5 ml/kg): Sigma Chemical Co., St. Louis, Missouri, USA] or NS-398 (Tocris Cookson Inc., Ellisville, Missouri, USA) at a dose of 5 mg/kg/day for 2 weeks (days 1–5 and 8–12). During this period, the right tibiae/fibulae were subjected to external mechanical loading [14–16] 3 h after injection on three alternate days per week (see the “External mechanical loading” section for details). We selected to use NS-398, rather than clinically available COX-2 selective inhibitors, to be able to compare the present data with those using a single injection of NS-398 and a single period of mechanical loading [9, 10], and thus, the dose and timing of injection were determined based on these previous studies [9, 10]. The left tibiae/fibulae were used as internal controls, as validated in the present model [16] and confirmed by others in the rat ulna axial loading model [17], and normal activity within the cages was allowed between external loading periods. On day 15, animals were euthanised and their left control and right loaded tibiae/fibulae collected for analysis of three-dimensional bone architecture. In the present study, ovariectomy was not performed because oestrogen withdrawal could modify the effects of COX-2 selective inhibitors on bone [13].

### External mechanical loading

The apparatus (model HC10; Zwick Testing Machines Ltd., Leominster, UK) and protocol for non-invasively loading the mouse tibia/fibula have been reported previously [14–16]. The tibia/fibula was held in place by a low level of continuous static preload (0.5 N for approximately 7 min), onto which a higher level of intermittent dynamic load (13.0 N) was superimposed in a series of 40 trapezoidal-shaped pulses (0.025-s loading, 0.050-s hold at 13.5 N, and 0.025-s unloading) with a 10-s rest interval between each pulse. Although a peak load of 12.0 N has been shown previously to induce significant osteogenic responses [18], a higher peak load (13.5 N) was selected in order to assess the effect of NS-398 on both lamellar and woven bone because a previous study had described different effects of NS-398 on lamellar and woven bone formation induced by a single loading episode [9]. It has been previously shown that this higher peak load results in loading-related woven bone formation in the cortical region of the proximal to middle tibiae and loading-related lamellar bone formation in the cortical region of the middle fibulae as well as in the trabecular region (secondary spongiosa) of the proximal tibiae [16]. Strain gauges attached to the proximal lateral tibial shaft of similar 19-week-old female C57BL/6 mice ex vivo showed that a peak load of 13.5 N engendered a peak strain of approximately 1,800 με [19].

### High-resolution micro-computed tomography analysis

Because mouse bone is small and the present axial loading-related osteogenesis is site specific, high-resolution micro-computed tomography (μCT; SkyScan 1172; SkyScan, Kontich, Belgium) with a voxel size of 5 μm was used to quantify three-dimensional bone architecture at precisely comparable sites of the loaded and contralateral control tibiae/fibulae as reported previously [15, 16, 18, 19]. Trabecular bone volume/tissue volume (BV/TV), trabecular number and trabecular thickness were measured in the secondary spongiosa of the proximal tibiae (0.05–1.00 mm distal to the growth plate). Cortical bone volume, periosteally enclosed volume, medullary volume and polar moment of inertia, a parameter of structural bone strength, were determined in 0.5-mm-long sections at four sites [25% (proximal), 37% (proximal/middle), 50% (middle) and 75% (distal) of bone length from its proximal end] in the tibiae and at the 50% (middle) site in the fibulae.

### Statistical analysis

All data are shown as the means and SEM. Statistical analysis was performed by one-way or two-way ANOVA using SPSS (version 17.0; SPSS Inc., Chicago, USA). *P* < 0.05 was considered statistically significant.

## Results

### Effects of NS-398 on body weight and bone length

There was no difference in body weight of mice treated with vehicle (day 1: 22.4 ± 0.4 g, day 15: 22.3 ± 0.3 g) or NS-398 (day 1: 23.0 ± 0.7 g, day 15: 22.6 ± 0.6 g). Bone length was similar in vehicle and NS-398-treated groups in the left control (17.8 ± 0.1 and 17.9 ± 0.1 mm, respectively) and right loaded (17.9 ± 0.1 and 17.9 ± 0.1 mm, respectively) tibiae and the left control (9.6 ± 0.1 and 9.8 ± 0.1 mm, respectively) and right loaded (9.7 ± 0.1 and 9.7 ± 0.1 mm, respectively) fibulae.

### Effects of NS-398 on trabecular and cortical bone

In trabecular bone of the proximal tibiae, NS-398 was associated with significant decreases in BV/TV and trabecular number, but not trabecular thickness, as shown in Table 1. In contrast, no effect of NS-398 was detected in cortical bone of the tibiae/fibulae.

**Table 1:** Trabecular and cortical μCT parameters in the left control and right loaded tibiae/fibulae in 21-week-old female C57BL/6 mice treated with vehicle or NS-398 (5 mg/kg/day, 5 days/week) for 2 weeks

	Vehicle + control	Vehicle + loading	NS-398 + control	NS-398 + loading	NS-398	*P* value loading	Interaction
Trabecular bone of the proximal tibia							
Bone volume/tissue volume (%)	17.5 ± 0.5	24.7 ± 0.9	16.4 ± 0.3	22.4 ± 0.4	**0.008**	**<0.001**	0.355
Trabecular number (mm^−1^)	3.30 ± 0.08	3.71 ± 0.10	3.10 ± 0.05	3.44 ± 0.05	**0.004**	**<0.001**	0.644
Trabecular thickness (μm)	52.9 ± 0.6	66.5 ± 0.9	52.7 ± 1.1	65.2 ± 1.0	0.441	**<0.001**	0.559
Cortical bone of the tibia							
Proximal site							
Bone volume (mm^3^)	0.403 ± 0.006	0.543 ± 0.010	0.411 ± 0.008	0.544 ± 0.011	0.642	**<0.001**	0.682
Periosteally enclosed volume (mm^3^)	0.713 ± 0.011	0.840 ± 0.012	0.741 ± 0.012	0.847 ± 0.014	0.170	**<0.001**	0.397
Medullary volume (mm^3^)	0.309 ± 0.007	0.297 ± 0.006	0.330 ± 0.007	0.303 ± 0.008	0.079	**0.013**	0.347
Proximal/middle site							
Bone volume (mm^3^)	0.378 ± 0.003	0.518 ± 0.010	0.386 ± 0.007	0.515 ± 0.010	0.761	**<0.001**	0.480
Periosteally enclosed volume (mm^3^)	0.614 ± 0.008	0.749 ± 0.008	0.626 ± 0.011	0.746 ± 0.010	0.638	**<0.001**	0.448
Medullary volume (mm^3^)	0.236 ± 0.007	0.230 ± 0.002	0.240 ± 0.006	0.231 ± 0.005	0.692	0.144	0.751
Middle site							
Bone volume (mm^3^)	0.297 ± 0.004	0.381 ± 0.004	0.309 ± 0.006	0.386 ± 0.010	0.208	**<0.001**	0.554
Periosteally enclosed volume (mm^3^)	0.483 ± 0.007	0.553 ± 0.006	0.495 ± 0.010	0.558 ± 0.011	0.319	**<0.001**	0.706
Medullary volume (mm^3^)	0.186 ± 0.004	0.171 ± 0.004	0.186 ± 0.005	0.172 ± 0.004	0.939	**0.002**	0.885
Distal site							
Bone volume (mm^3^)	0.274 ± 0.004	0.272 ± 0.004	0.280 ± 0.008	0.274 ± 0.006	0.474	0.475	0.747
Periosteally enclosed volume (mm^3^)	0.371 ± 0.005	0.373 ± 0.005	0.382 ± 0.009	0.381 ± 0.010	0.211	0.952	0.862
Medullary volume (mm^3^)	0.097 ± 0.002	0.102 ± 0.003	0.102 ± 0.002	0.107 ± 0.004	0.074	0.102	0.825
Cortical bone of the fibula							
Middle site							
Bone volume (mm^3^)	0.0523 ± 0.0009	0.0664 ± 0.0021	0.0511 ± 0.0006	0.0657 ± 0.0019	0.516	**<0.001**	0.878
Periosteally enclosed volume (mm^3^)	0.0587 ± 0.0014	0.0719 ± 0.0020	0.0562 ± 0.0005	0.0704 ± 0.0015	0.188	**<0.001**	0.712
Medullary volume (mm^3^)	0.0065 ± 0.0006	0.0054 ± 0.0003	0.0051 ± 0.0003	0.0048 ± 0.0006	0.054	0.160	0.527

Values are presented as the means±SEM (*n* = 8 in each group). Two-way ANOVA was used to compare groups. A *P* value of < 0.05 was considered statistically significant (in bold)

### Effects of NS-398 on trabecular and cortical bone’s response to mechanical loading

**Fig. 1:**
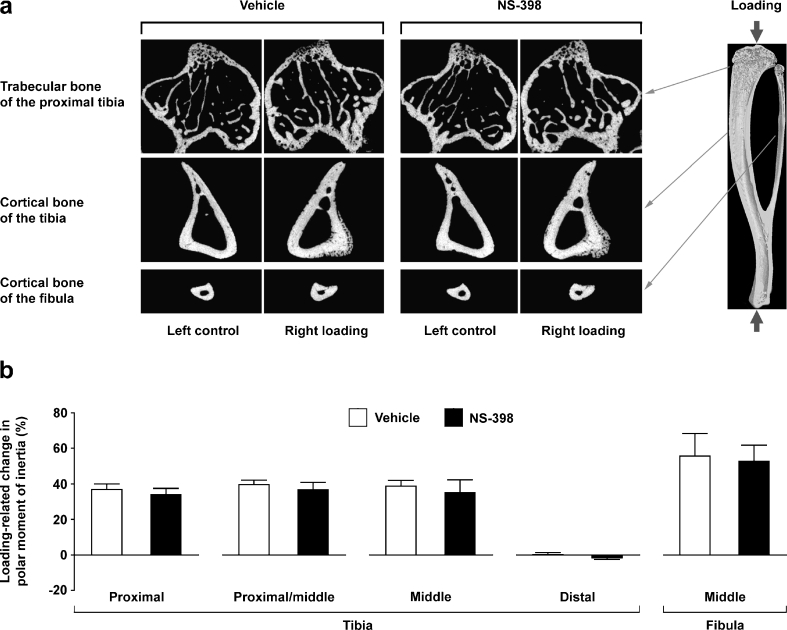
**a** Representative transverse μCT images of the left control and right loaded trabecular (0.5 mm distal to the growth plate) and cortical (37% site of the bone’s longitudinal length from its proximal end) bone in the tibiae and cortical bone (50% site of the bone’s longitudinal length from its proximal end) in the fibulae in 21-week-old female C57BL/6 mice treated with vehicle or NS-398 (5 mg/kg/day, 5 days/week) for 2 weeks. Note that woven bone formation is observed in cortical bone of the right loaded proximal/middle tibia, but not of the right loaded middle fibula. **b** Mechanical loading-related changes [(right loaded − left control)/left control] in polar moment of inertia, a parameter of structural bone strength, in 21-week-old female C57BL/6 mice treated with vehicle or NS-398 (5 mg/kg/day, 5 days/week) for 2 weeks. Values are presented as the means and SEM (*n* = 8 in each group). No significant difference was detected between vehicle and NS-398 groups by one-way ANOVA

In trabecular bone, mechanical loading significantly increased BV/TV, trabecular thickness and trabecular number (Table 1). Loading-related woven bone formation was not seen in the secondary spongiosa (Fig. 1a), as confirmed previously in the fluorochrome-labelled sections [16]. In cortical bone, the effects of mechanical loading were site specific; a loading-related increase in bone volume was obtained in the proximal and middle tibiae and middle fibulae, but not in the distal tibiae (Table 1). Consistent with a previous finding [16], in the proximal to middle tibiae, there was loading-related apparent woven bone formation while at the middle fibulae such a woven bone response was not observed (Fig. 1a). The loading-related increases in cortical bone volume and polar moment of inertia (Fig. 1b) were associated primarily with increased periosteally enclosed volume. No effect of NS-398 was observed on any of the loading responses at any site.

## Discussion

The present experimental design enabled evaluation of the effects of selective pharmacological inhibition of COX-2 by daily NS-398 injection on changes in three-dimensional bone architecture in trabecular as well as cortical bone induced by repeated episodes of mechanical loading. In control bones which received no more than normal functional mechanical loading, NS-398 slightly but significantly decreased trabecular BV/TV of the proximal tibiae. This would be compatible with a small reduction in bone mass of COX-2 deficient mice [11]. In bones that had been artificially loaded, COX-2 inhibition had no discernible effect on the loading-related lamellar or woven bone response in either trabecular or cortical compartments. As a result, NS-398 showed no influence on the loading-related increase in polar moment of inertia, a parameter of structural bone strength. Although there may be a potential small inhibitory effect of NS-398 on bone’s response to mechanical loading that could be detected only by histomorphometry, such an effect would not alter the conclusion of the present study.

The present data are consistent with the evidence from female mice lacking COX-2 [11], showing that bone adaptation to two consecutive days of mechanical loading does not require a functional COX-2 gene. The authors [11] suggested a compensatory effect of COX-1 in vivo, though this enzyme does not appear to be important for bone cells’ response to a single period of fluid flow in vitro [20]. If such compensation exists, it does not seem to be immediately available since in female rats a single injection of NS-398 reduces the cortical response to a single period of mechanical loading [9, 10]. The data we present here suggest that compensation for the pharmacological inhibition of COX-2 function does exist and can occur sufficiently swiftly to ensure that adaptive (re)modelling of trabecular and cortical bone to artificial mechanical loading over a 2-week period is not impaired.

The relevance of the present experiment in female mice to the human condition must take into account a number of differences in the two situations. Importantly, however, our experimental data of three-dimensional bone architecture analysed by high-resolution μCT are compatible with clinical evidence that women taking COX-2 selective inhibitors such as celecoxib and rofecoxib do not have lower hip areal BMD [13]. In contrast to women, the use of the COX-2 selective inhibitors is associated with lower hip areal BMD in men [13]. It remains to be elucidated whether there are sex differences in the effects of COX-2 inhibition on bone’s response to mechanical loading.

In conclusion, our present data demonstrate that in female mice pharmacological inhibition of COX-2 using daily NS-398 injection does not affect trabecular or cortical bone gain engendered by repeated periods of mechanical loading over a 2-week period. Should this experimental finding be translated into the clinical situation, it would suggest that in women long-term use of a COX-2 selective inhibitor does not impair the adaptive response of either trabecular or cortical bone to habitual mechanical loading and thus is not expected to contribute to bone loss by interference with this mechanism.

## References

[1] Suva LJ, Gaddy D, Perrien DS, Thomas RL, Findlay DM (2005). Regulation of bone mass by mechanical loading: microarchitecture and genetics. Curr Osteoporos Rep.

[2] Skerry TM (2008). The response of bone to mechanical loading and disuse: fundamental principles and influences on osteoblast/osteocyte homeostasis. Arch Biochem Biophys.

[3] Ozcivici E, Luu YK, Adler B, Qin YX, Rubin J, Judex S, Rubin CT (2010). Mechanical signals as anabolic agents in bone. Nat Rev Rheumatol.

[4] Bonewald LF, Johnson ML (2008). Osteocytes, mechanosensing and Wnt signaling. Bone.

[5] Price JS, Sugiyama T, Galea GL, Meakin LB, Sunters A, Lanyon LE (2011). Role of endocrine and paracrine factors in the adaptation of bone to mechanical loading. Curr Osteoporos Rep.

[6] Galea GL, Sunters A, Meakin LB, Zaman G, Sugiyama T, Lanyon LE, Price JS (2011). Sost down-regulation by mechanical strain in human osteoblastic cells involves PGE2 signaling via EP4. FEBS Lett.

[7] Pead MJ, Lanyon LE (1989). Indomethacin modulation of load-related stimulation of new bone formation in vivo. Calcif Tissue Int.

[8] Chow JW, Chambers TJ (1994). Indomethacin has distinct early and late actions on bone formation induced by mechanical stimulation. Am J Physiol.

[9] Forwood MR (1996). Inducible cyclo-oxygenase (COX-2) mediates the induction of bone formation by mechanical loading in vivo. J Bone Miner Res.

[10] Li J, Burr DB, Turner CH (2002). Suppression of prostaglandin synthesis with NS-398 has different effects on endocortical and periosteal bone formation induced by mechanical loading. Calcif Tissue Int.

[11] Alam I, Warden SJ, Robling AG, Turner CH (2005). Mechanotransduction in bone does not require a functional cyclooxygenase-2 (COX-2) gene. J Bone Miner Res.

[12] Kohrt WM, Barry DW, Van Pelt RE, Jankowski CM, Wolfe P, Schwartz RS (2010). Timing of ibuprofen use and bone mineral density adaptations to exercise training. J Bone Miner Res.

[13] Richards JB, Joseph L, Schwartzman K, Kreiger N, Tenenhouse A, Goltzman D (2006). The effect of cyclooxygenase-2 inhibitors on bone mineral density: results from the Canadian Multicentre Osteoporosis Study. Osteoporos Int.

[14] De Souza RL, Matsuura M, Eckstein F, Rawlinson SC, Lanyon LE, Pitsillides AA (2005). Non-invasive axial loading of mouse tibiae increases cortical bone formation and modifies trabecular organization: a new model to study cortical and cancellous compartments in a single loaded element. Bone.

[15] Moustafa A, Sugiyama T, Saxon LK, Zaman G, Sunters A, Armstrong VJ, Javaheri B, Lanyon LE, Price JS (2009). The mouse fibula as a suitable bone for the study of functional adaptation to mechanical loading. Bone.

[16] Sugiyama T, Price JS, Lanyon LE (2010). Functional adaptation to mechanical loading in both cortical and cancellous bone is controlled locally and is confined to the loaded bones. Bone.

[17] McKenzie JA, Silva MJ (2011). Comparing histological, vascular and molecular responses associated with woven and lamellar bone formation induced by mechanical loading in the rat ulna. Bone.

[18] Sugiyama T, Saxon LK, Zaman G, Moustafa A, Sunters A, Price JS, Lanyon LE (2008). Mechanical loading enhances the anabolic effects of intermittent parathyroid hormone (1-34) on trabecular and cortical bone in mice. Bone.

[19] Moustafa A, Sugiyama T, Prasad J, Zaman G, Gross TS, Lanyon LE, Price JS (2012) Mechanical loading-related changes in osteocyte sclerostin expression in mice are more closely associated with the subsequent osteogenic response than the peak strains engendered. Osteoporos Int. doi:10.1007/s00198-011-1656-4 10.1007/s00198-011-1656-4 PMC3304063 21573880

[20] Bakker AD, Klein-Nulend J, Burger EH (2003). Mechanotransduction in bone cells proceeds via activation of COX-2, but not COX-1. Biochem Biophys Res Commun.

